# Four Decades of Advancing Research on Adolescent Health and Informing Health Policies: The Health Behaviour in School-Aged Children Study

**DOI:** 10.3389/ijph.2025.1608136

**Published:** 2025-03-25

**Authors:** Oddrun Samdal, Colette Kelly, Wendy Craig, Joseph Hancock, Bente Wold, Leif Edvard Aarø, Joanna Inchley

**Affiliations:** ^1^ Department of Health Promotion and Development, University of Bergen, Bergen, Norway; ^2^ Health Promotion Research Centre, University of Galway, Galway, County Galway, Ireland; ^3^ Department of Psychology, Queen’s University, Kingston, ON, Canada; ^4^ School of Health and Wellbeing, University of Glasgow, Glasgow, Scotland, United Kingdom; ^5^ Department of Health Promotion, Norwegian Institute of Public Health (NIPH), Bergen, Norway

**Keywords:** HBSC, health behaviours, health, wellbeing, adolescent health, cross-national study, trends, health behaviour in school-aged children (HBSC)

## Abstract

The Health Behaviour in School-aged Children (HBSC) study is a large cross-national research study, conducted in partnership with the World Health Organization (WHO). The study has surveyed young people aged 11, 13 and 15 years every 4 years since the mid-1980s and has grown to include 50 countries across Europe, North America, and Western-Central Asia. Over the past 40 years more than 1.6 million students have participated. HBSC aims to advance understanding of adolescent health behaviours, health and wellbeing within social contexts, inform national and international health promotion policies and practice, and foster collaboration among researchers, policymakers, and practitioners. In this paper we share the history and development of the HBSC study covering: i) theory-driven and novel research impact, ii) unique long-term trends in adolescent health behaviours and perceived health and wellbeing, iii) methodological rigor to allow cross-national comparison, and iv) embedding youth involvement and maximizing policy impact.

## Introduction

Established in 1982, the Health Behaviour in School-aged Children (HBSC) study is a unique cross-national network aimed at understanding adolescent health behaviours and health across Europe, North America, and Western-Central Asia. Initially focused on health behaviours [1], it now covers a broad range of topics relevant to adolescent perceived health and wellbeing. Over 40 years, HBSC has become a key data source, providing critical evidence for national and international efforts to improve youth health outcomes, in partnership with the WHO Regional Office for Europe.

From its outset, HBSC has spearheaded the development of robust cross-national data collection procedures, providing a unique data infrastructure and resource for researchers and policymakers underpinned by a multidisciplinary international research network. Following the initiative of three researchers from England, Finland, and Norway, the HBSC study was formally established in 1982 [1]. The first data collection took place in 1983/84 in the three originating countries as well as Austria and later Denmark, with the intention of conducting surveys every second year among nationally representative samples of adolescents in schools. The second survey was undertaken in 1985/86 in 14 countries (Belgium is included as two separate regions, French-speaking and Flemish-speaking) and thereafter every 4 years with the following in 1989/90 in 16 countries. By the next survey in 1993/94 the number of countries had increased to 25, and this number almost doubled by 2021/22, with 50 network member countries of whom 46 undertook the survey. The most recent international data file from 2021/22 included more than 220,000 young people across the participating countries or regions, and over the past 40 years a total of more than 1.65 million students have been surveyed.

**FIGURE 1:**
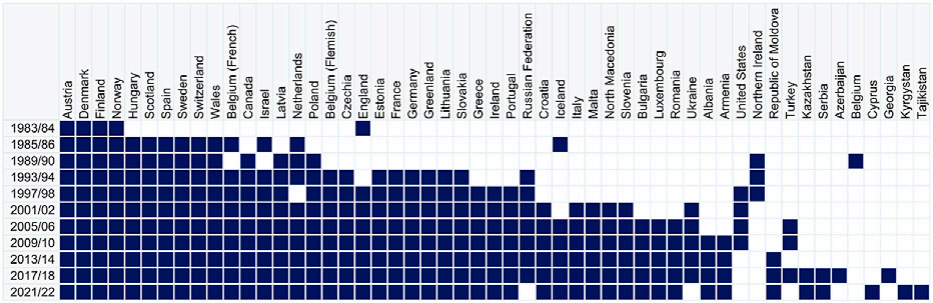
Member countries in the Health Behaviour in School-aged Children study across survey cycles from 1983/84 to 2021/22. Blue cells indicate study membership in each survey cycle. Countries are ordered by total number of survey participations (ascending from left to right), with alphabetical ordering within participation groups (Europe, North America, Western and Central Asia, 1983–2022).

Figure 1 shows the growth in the number of member countries across Europe, Western-Central Asia and North America, and when each country joined the HBSC network. In the first surveys, some countries (Belgium, England, France, Germany, Israel, Russia, and Scotland) were not able to obtain a fully nationally representative sample and instead sampled two to three regions in their country. From 2001/02, a new requirement was agreed whereby all countries had to have nationally representative samples for the three age groups sampled.

The age groups included are young people attending school aged 11, 13, and 15, which were chosen to represent the onset of adolescence (11 years), the challenge of physical and emotional changes (13 years), and the middle years when important life and career decisions are beginning to be made (15 years). Emphasis was given to expanding the study to include more countries, and specifically countries from middle and eastern Europe, where little information on adolescent health was available in the early 1980s. From the very beginning, collaboration was established with the World Health Organization’s European Office in Copenhagen [1]. This collaboration has played a crucial role in identifying and supporting new countries to join the network and has been essential in expanding the reach and impact of the study.

The HBSC cross-national comparisons across 50 countries offers many critical benefits such as understanding why and how certain phenomena differ across countries, which can provide insight into cultural, economic, and political issues [2]. In this way, HBSC provides a broader perspective on a particular topic or issue by identifying patterns, trends, and variations and testing the generalisability of findings. In addition, the cross-national data can provide policy and practice insights through an examination of how different countries address different issues. Moreover, cross country comparisons can test the applicability of theories across different contexts facilitating the development of more robust and universal theories. In this paper we highlight the development of the HBSC study over the last 40 years covering four main aspects: theory driven and novel research impact, providing long-term trends, ensuring methodological rigor, and youth involvement and policy impact.

### Theory-Driven and Novel Research Impact

Based on regular searches of publication using HBSC data, we have identified that over 1600 scientific peer reviewed journal articles have been published in English across the 40-year history of the study. Adding to that number are numerous peer-reviewed papers in national languages as well as book chapters and reports in many languages. Since 2001, HBSC data have been made available for researchers outside the network to encourage use and sharing of the data. This means that many of the published papers are written without the involvement of the HBSC network members and so likely the number of publications is much higher from this data set.

From its inception, the HBSC study aimed to advance new theoretical and methodological approaches to understanding and measuring adolescent health behaviours and wellbeing. As an example, it developed the widely used HBSC subjective health complaints scale (HBSC HCL) [3, 4]. The initial focus was on the role of socialisation in adolescent health behaviour and perceived health and wellbeing, addressing the interplay between individual characteristics and social/environmental contexts [1, 5], moving beyond the epidemiological focus of the 1980s.

Enabling novel scientific perspectives and emerging relevant topics when aiming to understand and explore adolescent perceived health has been a high priority for the HBSC. The majority of items used have been developed, piloted, and validated within the study, and today many are used by other international and national studies. Furthermore, emphasis has been given to apply theoretical frameworks when developing perspectives and items to be used in the study. Bronfenbrenner’s socio-ecological model [6, 7] has been an overarching guiding approach in combination with specific theories which aim to explain health behaviours or perceptions of health and wellbeing. Initially, HBSC explored the health compromising behaviour of smoking and its correlates [8], but soon after the health promoting behaviour of physical activity was included [9] to complement risk behaviours with health promoting aspects in line with the Ottawa Charter [10]. Guided by the socio-ecological model [7], emphasis in the 1990s was to understand how adolescents’ interactions with others in their main contexts such as the family, school, and leisure, related to their health behaviours and perceptions of health and wellbeing. Again, a novel emphasis was to frame the research questions with an asset perspective for the importance of promoting adolescent perceived health and wellbeing rather than primarily a risk perspective. Initially, focus was given to school as a setting. While previous research on schools had focused primarily on schools as a learning setting, HBSC started to explore the school as students’ work environment by adapting adult work environment theories [11] addressing autonomy, support, and expectations [12, 13]. In particular a focus was on understanding the school setting as an asset for thriving and healthy development. At the end of the 1990s the HBSC study started to more systematically explore the role of the family through items addressing structures of the family, family affluence, family support, and family activities [14, 15]. Similarly, the role of peer groups was studied through addressing identity formation, peer group norms, peer pressure, peer support, and participation in organised and non-organised activities [16, 17].

Throughout the 1990s the development of several other topics also started. While bullying in the school setting was included from the start using the Olweus’ [18] items, other aspects related to injuries and violence were developed and included at a time when little information on these behaviours were available [19, 20], and later perspectives on cyber-bullying were included [21]. The development of this topic area highlights how the survey responded to current emerging societal issues.

A highly used contribution from the HBSC study to the adolescent health research field is the development of the Family Affluence Scale (FAS) [22]. This measure was introduced in 1997 as a novel indicator of socio-economic status aiming to overcome the challenges of other measures asking adolescents to identify their parents’ education or occupation. The FAS measures ask students about the material resources within their home and family, which are more straightforward for adolescents to report. The measure is under continuous development and the fourth version is underway.

### Providing Long-Term Trends Alongside Continuing Commitment to Novelty

As the millennium approached, the HBSC study began examining time patterns in its data, revealing 40-year trends that provide unique insights into changes or stability in adolescent health behaviours and perceived health and wellbeing amid societal changes. These trends help explore secular changes over time, aligned with the Bronfenbrenner chrono system [7]. Recent examples have addressed national-level drivers of trends in adolescent mental health [23] and the impact of taxation policies on inequalities in soft drink consumption [24]. The long-term trends on several items have been explored by authors of this Supplement.

With growing recognition of the importance of the social determinants of health [25], HBSC continued its commitment to explore novel components that shed light on adolescents’ reporting of health behaviours and perceived health and wellbeing. As an example, the use of national level indicators, such as policies and economy, was introduced in the early 2000s to explain differences in individual behaviours within and between countries. The national gross domestic product (GDP) has been used as a country level indicator to understand social inequalities in adolescent health behaviours and health perceptions across countries [26, 27]. Similarly, the impact of smoking, alcohol and nutrition policies on adolescent health behaviours has been explored [28–30], as well as the impact of school systems on students’ perceptions of the school environment [31]. Moreover, the influence of country-level values, such as openness to change has also been studied [32]. In addition, Elgar et al. [33] examined how early exposure to income inequality in infancy and early childhood related to early involvement in bullying among adolescents, using the national GINI index for income inequality for every year spanning 35 years.

At the millennium shift, a new biological perspective in understanding the role of puberty and associated bodily changes on adolescent health and wellbeing was introduced [34, 35]. Building on this more recently a biopsychosocial or bioecological perspective [36] has been applied which recognises the importance of the dynamic interplay between biological, psychological and interpersonal factors and wider contextual factors in shaping health outcomes at the individual level [37, 38].

In the early 2000s HBSC also started to develop items to provide insights into adolescents’ sexual health behaviours, with a particular focus on romantic relationships and safe sex practices [39]. Likewise, the first attempts at capturing screen time use were initiated, initially with a focus on television viewing and computer use as leisure time activities but, more recently, items on adolescent social media use have been included [40] to better understand the role of digital technology and online communication on adolescents’ perceived health and wellbeing. Inclusion of new items responding to changes in theoretical perspectives and adolescents’ lives has all the time been balanced toward the needs for keeping space in the questionnaire to maintain items for the purpose of providing trends on adolescent health behaviours and perceived health and wellbeing.

## Methodology Development to Ensure Comparable Data

The HBSC has a standardised protocol specifying the theoretical framework, methods, questions, and data cleaning procedures, co-created by the HBSC network to ensure high data integrity and validity for cross-national comparison. Each survey round updates the protocol to include new theoretical perspectives and findings from pilot and validation studies. The most recent protocol [41] can be requested here, including just over 100 mandatory items included in the HBSC study covering health behaviours, perceived health and wellbeing and their social context correlates. Mandatory items are included in the questionnaires used for data collection by all countries. In addition, in-depth optional packages covering the same topics are developed and made available for internal use by member countries.

Over time, questionnaire development has sought to balance two purposes; i) maintain items across surveys to capture trends, and ii) explore new and current aspects of adolescents’ health behaviours, perceived health and wellbeing. To ensure that questions are translated appropriately to national languages a back-translation process is used emphasising translation of concepts.

The HBSC study uses a cluster sampling design, selecting all students in school classes to participate. The objective is to have 90% of the sample within 6 months of the mean age for the three age groups (11.5, 13.5, and 15.5 years) and to reach 1,500 students in each age group [41, 42]. In some countries, each age group corresponds to a single school grade, while in others a proportion of each age group may be found across grades. These national differences represent challenges in a cross-national study and need to be accounted for in the individual country’s sampling procedure [43].

### Youth Involvement and Cross-National Policy Impact

While individual HBSC country teams have involved adolescents for years, it was not until 2010 that the network formally embedded youth participation, based on Participatory Youth Research principles [44]. Initially, we explored which countries engaged with young people, the practices used, and the challenges faced. Today, youth participation is embedded in the network’s terms of reference, with an advisory group on Youth Engagement. This group has significantly supported country teams in involving adolescents in prioritising survey topics, analysing, interpreting, and disseminating findings. Adolescents from diverse countries have contributed to international protocols and reports, enhancing the study’s relevance and impact on policy and practice. Despite the challenges of involving youth across many languages and cultures, their insights are invaluable, providing an “insider” perspective on adolescent life. Together, we can futureproof the HBSC study to influence research, practice, and policy for future generations.

The HBSC study plays an essential role in shaping health promotion policies and practices targeting young people in each of the 50 participating countries as well as internationally. By providing comprehensive, cross-nationally comparative data, the study supports evidence-based decision-making and the development of targeted interventions to address current challenges in adolescent health, promote positive health outcomes and reduce health inequalities among young people [45].

Key findings from the international surveys are published every 4 years, through international reports presenting data by age, gender, and family affluence across a wide range of health and social indicators. A consistent theme in these reports has been the exploration of health inequalities, particularly gender and socio-economic disparities in adolescent health. These reports, published by the WHO Regional Office for Europe, are an essential resource for policymakers, researchers, and the wider adolescent health community. Following the most recent survey in 2021/22, the HBSC network adopted a new approach, moving away from a single comprehensive international report. Instead, the findings were released as a series of seven topic-focused international reports, allowing for more in-depth analysis of findings around specific topics and more targeted dissemination. Additionally, the HBSC study launched an online data browser (https://data-browser.hbsc.org/), which provides interactive access to the study’s findings. Users can explore and download charts and tables, organised by country, age group, and gender, allowing for detailed within-country and cross-national comparisons. This tool is designed to improve the accessibility and usability of HBSC data for researchers, policymakers, and public health professionals.

The HBSC study has made significant contributions to the development of various health promotion policies and initiatives at national and international levels. Initiatives such as the WHO’s Global Accelerated Action for the Health of Adolescents (AA-HA!) programme, for example, rely on HBSC data to guide countries in prioritising and implementing evidence-based interventions for adolescent health. HBSC data are also used to monitor European strategies on Child and Adolescent Health [46]. Furthermore, the study’s findings on substance use patterns and dietary behaviours have informed national policies aimed at reducing alcohol and tobacco consumption and soft drink consumption among adolescents, such as age restrictions, taxation measures, and public awareness campaigns. The HBSC study assists policymakers in identifying trends and emerging issues, ensuring that responses to the evolving needs of adolescents remain timely and relevant.

Representatives from the HBSC study also have been, and continue to be, directly involved in several international initiatives that aim to measure or report on adolescent health behaviours and perceived health and life satisfaction on a global scale. Recent examples are the Global Action for Measurement of Adolescent Health (GAMA), UNICEF Innocenti Report Cards, the OECD initiative on including adolescent wellbeing in their regular polls in member countries, and the WHO Global Physical Activity initiative that will publish on adolescents’ level of physical activity.

### Experienced Challenges

The expansion of the HBSC study included a number of challenges when aiming at standardising approaches across an increasing number of member countries. The rapid growth has substantially increased the diversity in the socio-cultural context of member countries making it more difficult to agree on mandatory questions that are applicable across all countries. Most of the new country teams are based in public health institutes financed by the Ministry of Health introducing funder requirements that are not typical for research funding. For example, for several of the countries the funding authorities have not accepted inclusion of questions on alcohol use, sexual behaviour, gender or sexual identity nor reporting of same sex parents in the questionnaire. To meet these needs the HBSC study has had to accept that countries can skip inclusion of mandatory questions when this is a funding requirement although that means we as a study lose out on providing important information on adolescents’ health behaviours, gender identity, and family context. Further, the increased diversity has made the questionnaire translation process more challenging. Finally, the increased diversity is also visible in the range of national requirements for ethical and GDPR approval from highly elaborated procedures to none. The latter is a particular challenge when publishing as most journals require documentation of such approvals, requiring us as a study to come up with solutions when they are not provided nationally.

### Future Directions

The HBSC study remains committed to advancing theoretical and methodological contributions by continuously enhancing our understanding of adolescent health behaviours and wellbeing. Guided by the socio-ecological model, the network’s researchers, from over 50 countries, leverage a diverse data set to address critical research questions. This large sample size and data richness have fostered international collaborations, producing research on global challenges like climate change and migration. Cross-national comparisons within the HBSC network have provided valuable insights, significantly contributing to academic knowledge and practical applications in policy and practice.

A future additional focus will be to better understand the impact of national and regional cultures on adolescent health outcomes. Given its many member countries, the HBSC study has a unique opportunity to explore how the macro system influences individual-level outcomes. Such investigation requires in-depth understanding of socio-cultural characteristics that may impact adolescents’ perceptions and is frequently attempted when aiming to explain differences in reported frequencies in individual health behaviours or perceived health and wellbeing. To achieve this goal, the HBSC study will actively seek collaboration with researchers within other relevant disciplinary fields, such as social anthropology, and employ databases including country-specific information on values and other characteristics, to provide better insights into country-level differences in individual level behaviours and health and wellbeing perceptions. In this way HBSC can enrich our understanding of cultural diversity whilst also enabling the identification of commonalities and universalities across diverse contexts.

Moreover, we are planning a large-scale validation study of new and existing items for the 2029/30 survey. Thereby we aim to maintain HBSC’s position at the forefront of contributing up-to-date knowledge of behaviours and social contexts of relevance for adolescent health and wellbeing.

Adolescents, defined as those aged 10–19 years, now constitute around a sixth of the world’s population [47], representing the largest generation of young people in history. Their perceived health and wellbeing in their different social contexts are vital to ensure a healthy development from childhood to adulthood. Further, with the disease burden shifting from childhood to adolescence, and recent advances in understanding of the critical neurobiological developmental processes which occur during adolescence, investment in public health and youth-friendly health services during this important stage of the life course is crucial. Early interventions of health promotion and prevention initiatives offer the best chance to make a difference to the lives of today’s young people and future generations.

### Conclusion

As we celebrate over 40 years of the HBSC study, the HBSC study remains committed to fostering a better scientific understanding of adolescent perceived health and wellbeing and informing the development of effective health promotion policies and practices worldwide. Through ongoing collaboration with international partners and stakeholders, HBSC will continue to address emerging challenges and contribute to the global effort to improve the lives of young people.
